# Genome-wide study of mRNA degradation and transcript elongation in *Escherichia coli*

**DOI:** 10.15252/msb.20145794

**Published:** 2015-01-12

**Authors:** Huiyi Chen, Katsuyuki Shiroguchi, Hao Ge, Xiaoliang Sunney Xie

**Affiliations:** 1 Department of Molecular and Cellular Biology, Harvard UniversityCambridge, MA, USA; 2 Department of Chemistry and Chemical Biology, Harvard UniversityCambridge, MA, USA; 3 Biodynamic Optical Imaging Center (BIOPIC), School of Life Science, Peking UniversityBeijing, China; 4 Beijing International Center for Mathematical Research (BICMR), Peking UniversityBeijing, China

**Keywords:** modeling, mRNA decay, RNAP elongation, RNA-seq, transcription

## Abstract

An essential part of gene expression is the coordination of RNA synthesis and degradation, which occurs in the same cellular compartment in bacteria. Here, we report a genome-wide RNA degradation study in *Escherichia coli* using RNA-seq, and present evidence that the stereotypical exponential RNA decay curve obtained using initiation inhibitor, rifampicin, consists of two phases: residual RNA synthesis, a delay in the interruption of steady state that is dependent on distance relative to the mRNA's 5′ end, and the exponential decay. This gives a more accurate RNA lifetime and RNA polymerase elongation rate simultaneously genome-wide. Transcripts typically have a single RNA decay constant along all positions, which is distinct between different operons, indicating that RNA stability is unlikely determined by local sequences. These measurements allowed us to establish a model for RNA processing involving co-transcriptional degradation, providing quantitative description of the macromolecular coordination in gene expression in bacteria on a system-wide level.

## Introduction

Descriptions of RNA synthesis and degradation are essential for understanding the dynamics of gene expression. The spatial localization of RNA in bacterial cells has been studied in detail by microscopy (Llopis *et al*, 2010; Nevo-Dinur *et al*, 2011). The kinetics of RNA synthesis have been measured directly in live *Escherichia coli* (*E. coli*) cells using fluorescently tagged RNA molecules (Golding & Cox, 2004). More traditionally, studies of RNA lifetimes of lacZ have contributed to describing the interaction of transcription, translation, and RNA degradation (Schwartz *et al*, 1970; Yarchuk *et al*, 1992). Studies were also performed within RNA transcripts, leading to the identification of unusually stable RNA segments (Von Gabain *et al*, 1983). However, in these cases, only one or few RNAs were studied, so it is difficult to identify global rules that can be applied genome-wide.

High-throughput techniques, like microarray, have increased the amount of information through genome-wide measurements, allowing researchers to observe the global behaviors for identifying the typical and the unusual in gene expression systems. The first microarray study of RNA lifetime in *E. coli* increased the number of measured RNA lifetimes by two orders of magnitude (Bernstein *et al*, 2002). The availability of such data allowed researchers to perform system-wide analysis and modeling (Bon *et al*, 2006; Deneke *et al*, 2013). Selinger *et al* (2003) designed a microarray using multiple probes along operons to study degradation at sub-genic resolution, yielding the insight that 5′ ends are generally less stable than 3′ ends. High-throughput sequencing technology that has emerged in recent years allows researchers to study nucleic acids with even higher base resolution. One of its applications is RNA sequencing (RNA-seq), which has been successfully used in genome-wide studies to investigate details in the mechanisms of processes like RNA polymerase transcription, and mRNA splicing (Ameur *et al*, 2011; Churchman & Weissman, 2011; Taggart *et al*, 2012). RNA-seq has been used to study RNA degradation in *Bacillus cereus* and *Bacillus subtilis*, although with limited or no time resolution (Kristoffersen *et al*, 2012; Liu *et al*, 2014). We now apply RNA-seq to the study of the kinetics of RNA dynamics to obtain high-resolution data genome-wide so that we can further understand the interplay between degradation and synthesis.

The RNAP initiation inhibitor, rifampicin, has been used in genome-wide studies of RNA degradation (Bernstein *et al*, 2002; Selinger *et al*, 2003). The degradation of RNA is observed and measured after synthesis is inhibited. While rifampicin binds all free RNAPs, it does not affect RNAPs that are already bound and transcribing on the DNA, resulting in residual RNA synthesis (Pato & Von Meyenburg, 1970). The effect of such residual RNA synthesis upon RNA decay has already been indirectly inferred from the observed delays in RNA decay (Von Gabain *et al*, 1983; Yarchuk *et al*, 1992; Chow & Dennis, 1994) and modeled mathematically (Deneke *et al*, 2013), although others have attributed the delay to rifampicin permeability (Llopis *et al*, 2010). If the resolution of the measurement is high enough to capture such a distance-dependent delay and it is proved to be caused by residual synthesis, then RNA elongation rates can be measured genome-wide.

In this study, we obtained details of global RNA dynamics in exponentially growing and stationary phase *E. coli* cells using RNA-seq, with sub-minute time resolution to allow observation of RNA synthesis and degradation. We observed, similar to previous studies (Chow & Dennis, 1994; Llopis *et al*, 2010; Deneke *et al*, 2013), that there is a delay before RNA abundance decays after the addition of rifampicin in both growth conditions. We show through a direct experiment that elongating RNAPs are primarily responsible for this delay. As a result, we extracted RNAP elongation rate and RNA degradation rate simultaneously from our high-resolution data. The fitted exponential lifetimes for RNA degradation at all positions of a transcript are similar for most mono-cistronic and poly-cistronic RNAs. RNA dynamics in stationary phase *E. coli* exhibit the same trends as in exponentially growing cells, suggesting that any coordination between transcription and degradation remains essentially unchanged despite changes in transcription initiation rates. Finally, we revisited the meaning of the observed exponential decay of RNA and proposed that co-transcriptional degradation is prevalent among RNAs, underscoring the interlinked nature of transcription, translation, and RNA degradation.

## Results

### Simultaneous measurement of RNA chain elongation and degradation genome-wide using rifampicin

**Figure 1:**
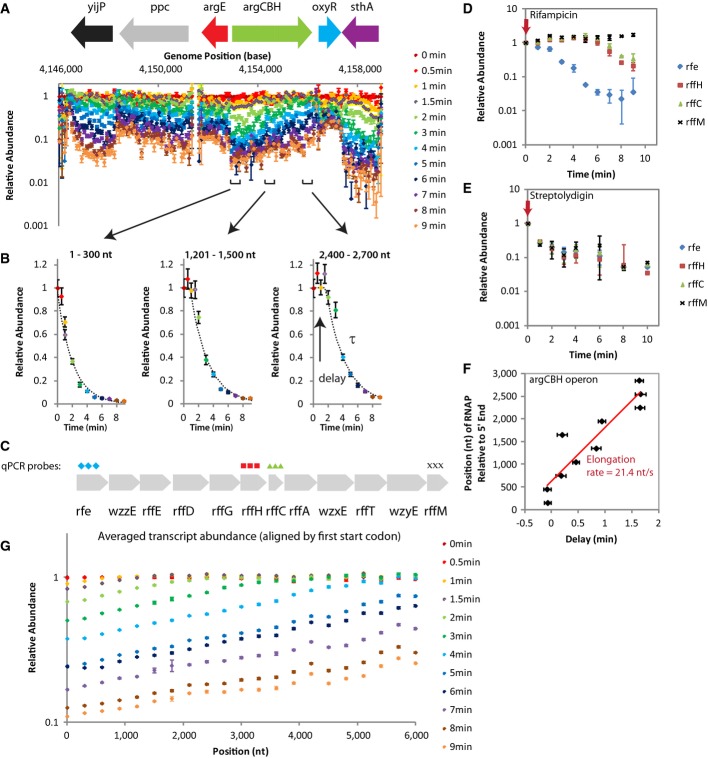
RNA-seq data allow detailed study of RNA synthesis and degradation genome-wide

RNA-seq was used to measure the abundance of mRNA in *E. coli* cells growing exponentially (OD_600_ = 0.3) at various time intervals after treatment with rifampicin, an initiation inhibitor of RNAP. The data were segmented into 300-nucleotide bins according to transcription unit annotation (Keseler *et al*, 2013) and include both mRNA and non-coding RNA (Supplementary Tables S1 and S2). *In vitro* synthesized RNAs, used as a non-degrading control, were added at defined concentrations to the bacterial lysate and used to normalize relative abundance within time points (Supplementary Fig S1). The abundance of RNA at different positions is further normalized with respect to the data at the zero-minute time point. After the normalization, our genome-wide dataset revealed that there is less RNA on the 5′ ends compared to the 3′ ends of RNA transcription units at subsequent time points (Fig1A). This difference between the 5′ and 3′ ends was previously noted by Selinger *et al* (2003), who concluded that there was a difference in 5′ and 3′ stabilities on RNAs. This observation was thought to support the idea that degradation generally happens in a 5′ to 3′ direction (Selinger *et al*, 2003). We confirmed the RNA-seq measurements by quantitative PCR (qPCR) (Supplementary Figs S2 and S3).

RNA abundance data over time of exponential phase MG1655 cells show trends correlating with direction of RNA synthesis. Representative normalized RNA abundance measured by RNA-seq (one experiment) for each time point is shown with corresponding genome annotation and root N error. Segments where the data exhibit a similar degradation pattern correspond with the annotated operons. The small RNA, oxyS, has been omitted from the genomic annotation due to its small size.

RNA abundance data from segments of the argCBH operon plotted against time reveal a delay in degradation. The data were fitted to extract the delay before RNA abundance decay, and the exponential lifetime, τ.

4 qPCR probes were designed against the 12-kb rfe-wzzE-rffEDGHCA-wzxE-rffT-wzyE-rrfM transcript for the experiment shown in (D) and (E), which confirms that elongating RNAPs are responsible for the delay before RNA abundance decays.

qPCR experiment in exponential phase AS19 cells treated with rifampicin, an RNAP initiation inhibitor, replicates the trend observed in RNA-seq data for exponential phase MG1655 cells treated with rifampicin. RNA in the most 5′ position, rfe, declines in abundance immediately. Mid-transcript positions (rffH and rffC) maintained steady-state levels of RNA abundance for ˜6 min before showing degradation. The most 3′ position, rffM, maintained steady-state levels of RNA for the entire duration of the experiment. Data are from two biological samples with duplicates and error bars show s.d.

qPCR experiment in exponential phase AS19 cells with streptolydigin, an RNAP elongation inhibitor, does not replicate the trend observed in RNA-seq data for exponential phase MG1655 cells treated with rifampicin. RNA abundance at all probe positions declines immediately after streptolydigin addition. Data are from two biological samples with duplicates and error bars show s.d.

The RNAP elongation rate, assumed constant and measured at 21.4 nt/s (s.e. = 0.5 nt/s), on the argCBH operon is extracted from the delay in degradation along the operon.

Averaged genome-wide view of data. All RNAs were aligned by their first start codon and averaged to obtain a genome-wide view of RNA abundance. The trends observed in (A) are reproduced on the average genome-wide scale, suggesting that they are typical.

A closer examination of the data revealed that the differences in 5′ and 3′ abundances are due to a delay in the appearance of degradation (Fig1B), while the exponential decay was similar across the operon. This argues against the existence of differential lifetime along the transcript. Instead, it suggests that transcription elongation is visible in the data. Similar decay curves have been reported in the literature and are thought, but not confirmed, to be the result of elongating RNAPs (Chow & Dennis, 1994). To check for the contribution of elongating RNAPs, we compared the RNA degradation measured by adding an RNAP elongation inhibitor, streptolydigin, to RNA degradation measured by adding rifampicin. By qPCR, we observed that the 5′-end of our 12-kb transcript (Fig1C and D) showed degradation immediately after the cells were treated with rifampicin, similar to the RNA-seq data. Furthermore, the downstream segments had stable RNA abundance for an amount of time proportional to their distance from the 5′ end (Fig1D). These positional differences in the onset of declining RNA abundance were not apparent when the cells were treated with streptolydigin (Fig1E), confirming that the products of elongating RNAPs are visible in the rifampicin data. The 5′ to 3′ differences in RNA degradation are caused through RNA synthesis by RNAPs that had initiated transcription prior to rifampicin addition.

Therefore, we fitted our data differently from previous papers by including a delay before the exponential decay (Fig1B). Each delay represents the time when the last average RNAP went through the transcript segment. Because most promoters in bacteria are weak, there is usually one or no RNAP transcribing each transcription unit per cell (Bon *et al*, 2006; Proshkin *et al*, 2010). The last average RNAP is thus representative of RNAPs in their physiological condition. Then, linear fitting of the delay time across the length of the RNA was performed to extract the elongation rate (Fig1F). Unlike previous methods of measuring RNAP elongation rates that relied on specific small molecule induction of a single RNA (Rose *et al*, 1970; Vogel & Jensen, 1994), our approach, using RNA-seq and rifampicin, is basically capable of measuring the native elongation rate of all actively synthesized RNAs in the cell.

The 5′ to 3′ differences in RNA abundance are general across the genome, evident from the averaged genome view (Fig1G). It also confirms that the averaged delay before start of RNA degradation scales proportionally to the distance from the 5′ end and that the relative abundance on average decreases at a similar rate from the 5′ end to the 3′ end. The expected delay of rifampicin action is < 30 s (Pato & Von Meyenburg, 1970).

### Global behavior of RNA degradation

**Figure 2:**
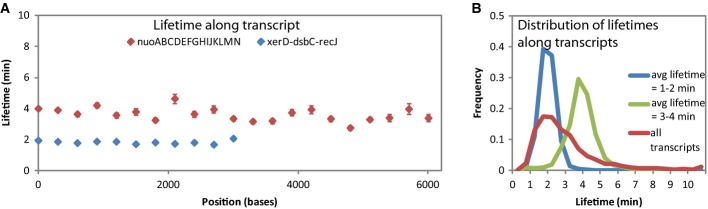
Lifetime is generally constant across RNAs

The RNA lifetimes extracted from our fitting, which corrects for the delay in RNA degradation, showed that all positions along an RNA generally have a lifetime similar to other segments on the same transcript (Fig2). For all mono-cistronic and most poly-cistronic RNAs that we measured, consecutive segments along a transcript have lifetimes that are within twofold of each other (Supplementary Table S1). We found examples of poly-cistronic transcripts that had different lifetimes across the operon. In agreement with a previous report, we found that the malEFG RNA has segments with different lifetimes (Newbury *et al*, 1987) (Supplementary Table S3). We also found five other poly-cistronic RNAs with different segment lifetimes (> twofold difference) that have not been reported in the literature (Supplementary Table S3). We also note that in a few cases, all segments of some very long transcripts, smtA-mukFEB and hybOABCDEFG, had a single exponential lifetime that was much shorter than the time estimated for the synthesis of the full-length transcript from the measured elongation rate, which suggests co-transcriptional degradation for at least these transcripts (discussed later).

The lifetime along all segments of 3,371-nt transcript, xerD-dsbC-recJ, is ˜2 min (s.e. shown). The lifetime along most segments of the 14-kb nuo operon is ˜4 min (s.e. shown).

The distribution of lifetimes of all segments from transcripts longer than 600 nt with an average lifetime of 1–2 min (blue) and 3–4 min (green) is different from each other and more narrow compared to the distribution of lifetimes of all transcripts (red).

**Figure 3:**
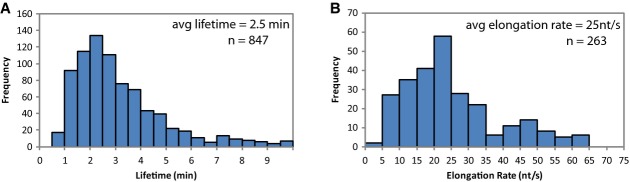
Distributions of lifetimes and elongation rates measured for exponentially growing cells

We average the lifetimes of the 300-nt segments along each transcript and report these lifetimes (Fig3A; Supplementary Table S4). Our measurements gave an average lifetime of 2.5 min, shorter than the previous genome-wide microarray measurements which have an average lifetime of 6 min for genes, probably because of our correction for residual RNAP activity (Bernstein *et al*, 2002; Selinger *et al*, 2003). The distribution of lifetimes of operons and genes is similar (Supplementary Fig S4). The previously reported correlation between gene function and lifetime (Bernstein *et al*, 2002; Selinger *et al*, 2003) was not observed in our data (Supplementary Table S5).

The lifetimes of 847 transcripts were measured, with an average lifetime of 2.5 min (standard deviation 2.5 min).

The elongation rates for 263 transcripts longer than 1,200 nt were measured. The average is 25 nt/s (standard deviation 14 nt/s).

### RNA chain elongation rates genome-wide

We were able to measure RNA chain elongation rates for 263 transcripts of > 1,200 nt length that have enough data for analysis, producing a survey of native mRNA chain elongation rates (Fig3B; Supplementary Table S6). Our average elongation rate of 25 nt/s agrees with previous measurements of native mRNA transcription elongation rates. The trp operon was previously measured at 27 nt/s, and bulk mRNA elongation rates were estimated at 29 nt/s (Manor *et al*, 1969; Rose *et al*, 1970). A few of the mRNA transcription rates are in the range of speeds typically associated with ribosomal RNA transcription (]70 nt/s) and may be the consequence of regulation by transcriptional elongation factors (Roberts *et al*, 2008). Many other elongation rates have been measured by cloning the gene of interest into a plasmid behind an inducible promoter (Vogel & Jensen, 1994; Proshkin *et al*, 2010). However, it has shown that plasmid-based measurements of elongation rates can be changed by varying the concentration of IPTG (Epshtein & Nudler, 2003). Our measurements rely on native RNAP initiation rates, which are directly regulated by the cell.

### Comparison of exponential and stationary phase RNA dynamics

**Figure 4:**
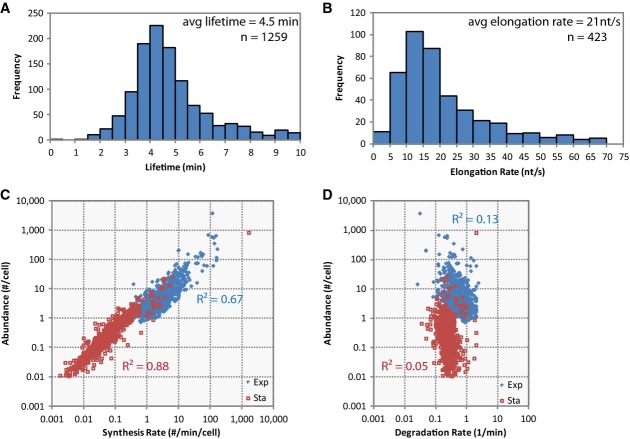
Comparison of stationary phase measurements with exponential phase measurements

We also performed measurements for cells growing in stationary phase (OD_600_ = 1.7). We found that lifetimes were still generally constant across transcripts, and averaged ]4.5 min, with a smaller deviation than for exponential phase lifetimes (Fig4A; Supplementary Tables S2 and S7). Elongation rates were slightly slower than those measured for RNAs expressed in exponential phase (Fig4B; Supplementary Table S8). The spike-in RNAs used for normalization also allowed us to estimate that the average copy number of measured RNA was ]50 times lower in the stationary phase cells. Furthermore, knowing both steady-state abundance (time zero in our measurement) and RNA lifetimes allowed us to calculate synthesis rates, which revealed that the change in RNA abundance was better explained by changes in transcription initiation rates (*R*^2^ = 0.67–0.88) than in lifetimes (*R*^2^ = 0.05–0.13) (Fig4C and D).

Stationary phase average lifetime is 4.5 min (s.d. = 2.4 min). The lifetimes of 1,259 transcripts in stationary phase *Escherichia coli* were measured. Relative to exponential phase lifetimes which have a standard deviation as large as the mean (2.5 min/2.5 min = 1), the distribution of lifetimes in stationary phase is narrow (2.4 min/4.5 min = 0.53).

Stationary phase average elongation rate is 21 nt/s (s.d. = 14 nt/s). The elongation rates for 423 transcripts longer than 1,200 nt for stationary phase *E. coli* were measured.

RNA abundance can be explained by RNA synthesis rate for both exponential and stationary phase cells. RNA abundance and transcription initiation rates correlate well for the exponential phase measurements (*R*^2^ = 0.67) and have an even stronger correlation in stationary phase (*R*^2^ = 0.88).

RNA abundance is poorly correlated to degradation rate (1/lifetime) in exponential and stationary phase *E. coli*. Degradation rates do not correlate well with RNA abundance in exponential (*R*^2^ = 0.13) and correlates more poorly in stationary phase (*R*^2^ = 0.05), in contrast to the correlation of RNA abundance with synthesis rates.

### A dynamic model incorporating transcription, translation, and RNA degradation in *Escherichia coli*

**Figure 5:**
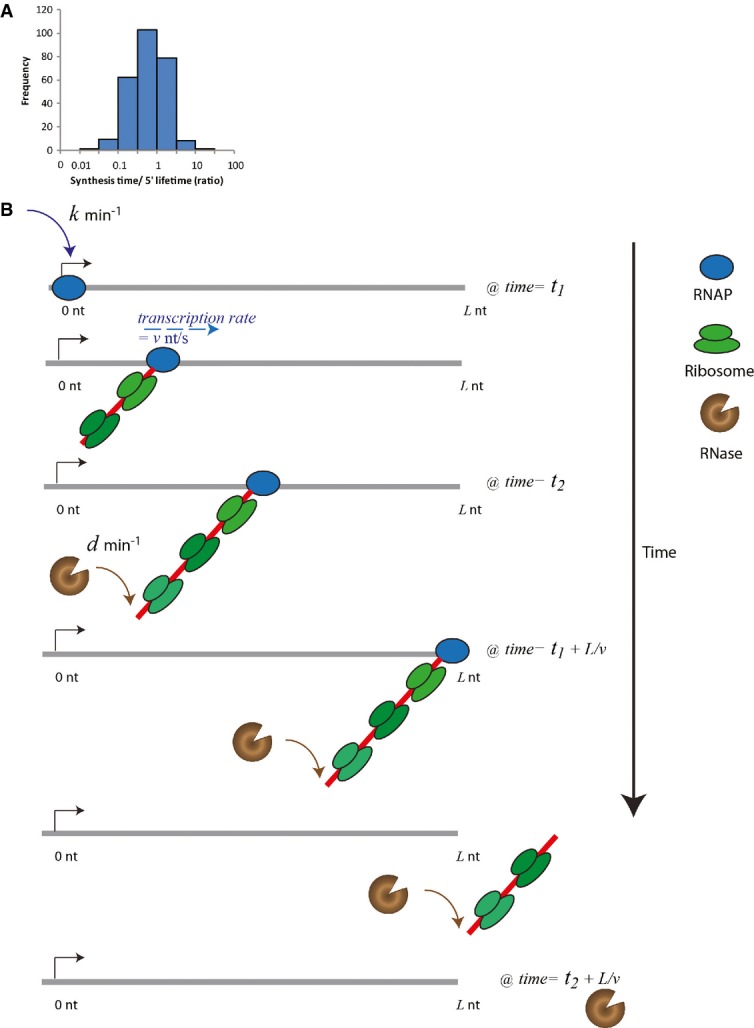
Co-transcriptional degradation of RNA

We observed that some transcripts, like the smtA-mukFEB and hybOABCDEFG operons mentioned earlier, are so long that the amount of time needed to synthesize the full-length RNA is longer than the lifetime of the 5′ end of the transcript. In other words, the 5′ end is degraded before the 3′ end is synthesized, which is called co-transcriptional degradation. The degradation of the 5′ end of an RNA before the synthesis of its 3′ end has previously been shown for the trp and lac operons (Morikawa & Imamoto, 1969; Morse *et al*, 1969; Cannistraro & Kennell, 1985). However, there has not been a systematic study of co-transcriptional degradation. For the 263 transcripts with a measured elongation rate in our dataset, we found that 88 (33%) in exponential phase have a synthesis time that is longer than the lifetime of their 5′ end (synthesis time/lifetime > 1) (Fig5A; Supplementary Table S9), suggesting that co-transcriptional degradation is potentially common.

88 out of the 263 RNAs with a measured elongation rate in exponentially growing cells have a synthesis time (estimated from the length of the RNA and the elongation rate) that is longer than the lifetime of the 5′ end.

Model of temporal coordination of co-transcriptional degradation on RNA transcript of length *L* explains degradation curves and how synthesis time can be longer than lifetime. Initially at *T *= *t*_1_, RNAP binds to the genome with a single rate of transcriptional initiation, *k* (1/min), and proceeds to generate the RNA transcript with a constant elongation rate, *v* (nt/s). At *T *= *t*_2_, before full-length RNA is generated, RNase binds to the 5′ end in a rate-determining step with rate *d* (1/min), which is affected by the competition between ribosome and RNase binding. Any subsequent binding is not rate limiting. The RNase cannot degrade the RNA more quickly than the movement of the last ribosome (dark green) on the transcript. At *T *= *t*_1_ + *L*/*v*, the RNAP generates the end of the RNA transcript. Because the RNase has already bound and started to degrade the transcript, the transcript length is less than *L* nts at *T *= *t*_1_ + *L*/*v*.

The coordination between transcription, translation, and degradation in the cell is generally described. Transcription is initiated by RNAP binding, and the RNAP proceeds at a constant speed. As soon as the ribosome binding site is synthesized, ribosomes can bind the mRNA to start translation. Enzymes with RNase activity also target the 5′ end of the mRNA and compete with ribosomes to bind and degrade. The exact temporal coordination is, however, not well understood.

To better understand the prevalence of co-transcriptional degradation, we start with the simplest single compartment model. Although some RNases are limited by their membrane localization, the quantitative information needed to build a more complicated model is not yet available. Hence, we use a minimal model, which is consistent with our quantitative data; hence, the following scenario based on our experimental observations is modeled: The initiation of transcription occurs with a single rate-limiting step with rate *k* and the elongation speed, *v*, is assumed to be uniform across the same transcript, which is consistent with our data. Since the lifetimes of RNA segments do not vary along the same transcript, we assume the binding of RNase at the 5′ end of the transcript, competing with ribosomes, serves as the single rate-limiting step with rate *d* for the whole RNA degradation process. This assumption does not distinguish between the two possible 5′-dependent degradation enzymes (Supplementary Information). We further assume that RNase that is bound to the transcript closely trails behind ribosomes, rapidly cleaving the ribonucleic acid after the last ribosome molecule, instead of being able to cut between ribosomes (Fig5B) (Schneider *et al*, 1978). Although mRNA can be cleaved in between ribosomes to rescue stalled ribosomes, the occurrence of such an event is low, 0.4%, and does not affect our model (Moore & Sauer, 2007). Lastly, we assume the elongation velocity of the ribosome on the transcript is similar to that of the RNAP, which has been observed experimentally (Proshkin *et al*, 2010). This ensures that the lifetime of the 5′ and 3′ ends is similar.

Next, we consider two different mechanisms: One is co-transcriptional degradation, which allows RNase to bind RNA at/near the 5′ end at any time after RNA synthesis has started (Fig5B), while the other one is post-transcriptional degradation, which only allows RNase to bind at/near the 5′ end only after the entire transcript is completely synthesized.

The co-transcriptional degradation model is solved by a delayed single exponential decay curve (Supplementary Information). This means that RNA abundance exhibits a position-dependent delay followed by single exponential decay at all segments of an RNA, similar to our experimental observations. This is in contrast to the post-transcriptional model, which has a more complex solution. It has a delay (equilibrium), followed by linear decay and then finally an exponential decay. Like in the co-transcriptional model, the delay is dependent on the distance of the segment from the 5′ end, while the linear decay is due to waiting for RNA synthesis to complete before degradation can commence (Supplementary Information).

In the fastest case of post-transcriptional degradation, that is, the binding rate *d* of RNase is extremely high, RNA instantaneously degrades the moment it is completely synthesized, the degradation of the RNA abundance at the 5′ end is exactly linear (Supplementary Information). When the binding rate *d* is finite, the decay curve of the RNA abundance at the 5′ end should always be slower than that of instantaneous degradation. Therefore, in such a post-transcriptional case, the RNA lifetime at the 5′ end should be greater than the synthesis time for the whole transcript.

On the contrary, when the ratio of synthesis time/lifetime > 1, there are moments where the 5′ end RNA abundance is lower than that expected from the fastest post-transcriptional decay mechanism, for example, the smtA-mukFEB operon (Supplementary Fig S5). It indicates that the degradation of these transcripts is co-transcriptional. Even though we do not have a synthesis time/lifetime ratio larger than 1 for all observed RNAs to identify co-transcriptionally degraded RNAs, by Occam's Razor, we think that co-transcriptional degradation is a simple mechanism that can explain observed degradation patterns.

We caution that a co-transcriptional degradation mechanism does not imply that every single produced RNA in the cell is being synthesized and degraded simultaneously. Both ribosome binding, which is proposed to be protective (Schneider *et al*, 1978), and RNase binding, which initiates degradation, are stochastic events on the molecular level. While there are instances where the RNase binds before the RNAP is done synthesizing the RNA, there are other instances when ribosomes happen to consecutively bind so that the full-length RNA is synthesized before RNase has a chance to bind and initiate degradation. From our model, we can calculate the probability of having a transcript that is degraded before the full transcript has been synthesized to be 1-e^*dL*/*v*^ (Supplementary Information), which is dependent on the ratio of RNA synthesis time to RNA lifetime. For transcripts with a synthesis time/lifetime (*dL*/*V*) > 1, the probability of each RNA molecule being co-transcriptionally degraded is > 60%. If the co-transcriptional degradation model holds genome-wide, even for transcripts that have a synthesis time/lifetime < 1, we can still calculate that a sizeable fraction of RNA that is simultaneously being synthesized and degraded. For instance, with a synthesis time/lifetime of 0.5, 40% of RNAs are degraded before completely being synthesized.

The identification of more transcripts, besides the lac and trp operons, that are likely to be co-transcriptionally degraded has increased the amount of evidence supporting genome-wide co-transcriptional degradation. Moreover, co-transcriptional degradation likely does not happen exclusively to very long transcripts as in the literature. The generality of constant lifetime across the same transcript implies that RNAs are at least degraded in a 5′ to 3′ direction. It would thus follow that determinants of RNA lifetime can be expected to preferentially exist on the 5′ proximal end of the transcript. This conclusion ties in well with the observation that the 5′ end contains important determinants that regulate RNA lifetime (Arnold *et al*, 1998).

## Discussion

The fundamental observation that RNA decays exponentially with a single lifetime, established in early experiments on a few RNAs, indicates the existence of a single rate-determining step (Mosteller *et al*, 1970; Schwartz *et al*, 1970). This rate-determining step is thought to initiate degradation (Carpousis, 2007; Belasco, 2010). In our study, we find that segments within the same transcript genome-wide share a similar rate constant (Fig2). This suggests that the effect of the rate-determining step is shared by all parts of the RNA and that transcription, translation, and degradation work together in a defined manner to achieve such an outcome genome-wide. While our work does not comment on the specific mechanism of the decay pathway, it provides limits to the models of RNA degradation on a genome-wide scale, which has not yet been achieved in the literature. Following the example of earlier studies of RNA degradation that have been more holistic, we integrate the kinetics of transcription, translation, and degradation into a mathematical model that predicts our RNA-seq results. This detailed modeling of RNA dynamics in *E. coli* supports the generality of our assumptions, which are based on data obtained from gene-specific studies.

A consequence of exponential decay that has been only partially addressed in the literature is that RNA can be broken down at any time after the initiation of synthesis, even before the entire transcript is synthesized (Morikawa & Imamoto, 1969; Morse *et al*, 1969; Cannistraro & Kennell, 1985). This means that RNAs can be co-transcriptionally degraded. This is counterintuitive since full-length RNA is the major observed mRNA species in the cell (Llopis *et al*, 2010), which has been shown to diffuse and localize to different parts of the cell in some cases (Nevo-Dinur *et al*, 2011). Moreover, the existence of subcellular compartmentalization, with separate zones of DNA, ribosomes, and RNases that RNA has to diffuse through, makes co-transcriptional degradation less plausible (Mackie, 2013). However co-transcriptional degradation is a stochastic process, not a deterministic one, which allows full-length and partially degraded mRNA to coexist via the same mechanism. In our work, we proposed a dynamic model to distinguish the mechanisms of co-transcriptional and post-transcriptional RNA degradation that allows us to estimate the distribution of full-length and already degraded RNAs before the completion of transcription for a single transcript species.

In summary, RNA degradation was measured with high time and base resolution in exponentially growing and stationary phase cells, leading to the clear observation of a lag in RNA degradation that is dependent on the distance from the 5′ end after the addition of transcription inhibitor, rifampicin. This allowed us to confirm simultaneous observation of transcription elongation and degradation, resulting in a first survey of native RNAP elongation rates, and more accurate extraction of RNA lifetimes along RNAs genome-wide. The observed constant exponential lifetime along transcripts suggests 5′ to 3′ directed RNA degradation and supports the mechanism of co-transcriptional degradation for each transcription unit. Taken together, the parameters provided by our measurement highlight the tight coordination between transcription and RNA degradation in *E. coli* cells.

## Materials and Methods

### Cell growth and preparation

*Escherichia coli* strains K12 MG1655 or AS19 (gift of Peter Nielson, University of Copenhagen, Denmark) were grown in LB-Miller (EMD) at 30°C with shaking at 250 rpm. Overnight cultures were diluted 1:250 into fresh media and grown to OD_600_ = 0.3 for the exponential phase sample and OD_600_ = 1.7 for the stationary phase sample. The doubling time during steady-state growth in exponential phase was ]40 min.

Rifampicin (Sigma R7382, previously R5777), dissolved in DMSO, was added to MG1655 and AS19 cultures at a final concentration of 500 ng/μl, and aliquots of the cultures were quenched in 10% volume of cold 9:1 ethanol:phenol solution at specified time points. The cells were pelleted and washed with cold PBS before storage at −80°C.

Streptolydigin (ChemCon GmbH) was dissolved in water and added to AS19 cultures at a final concentration of 100 ng/μl.

### RNA purification

Bacterial pellets were thawed on ice. Cells were first resuspended and incubated in 1 mg/ml of lysozyme (Sigma) prepared in T.E. buffer and then completely lysed by the addition of 1 volume cell lysis buffer (Puregene). Spike-in RNAs were introduced at this stage.

The samples were extracted with acidic phenol/chloroform (Sigma) 2–4 times and then once with chloroform:isoamyl alcohol (24:1, Sigma). The RNA was then precipitated with the addition of 1/10 volume 5 M NaCl and 1 volume isopropanol on ice or in −20°C. RNA was pelleted and washed twice with 70% ethanol, resuspended to 100–200 ng/μl in nuclease-free water (Ambion or Cellgro), then digested with DNaseI (NEB) to remove contaminating DNA. The RNA was then purified as described. After resuspending in water, the RNA was used immediately.

### RNA-seq protocols and rRNA removal

rRNA was removed by Epicentre's Ribo-Zero Kit (for gram-negative bacteria) following manufacturer's protocol. RNA was finally collected by RNA Clean-up and Concentration column (Zymo Research).

The RNA was fragmented and prepared for Illumina sequencing as previously reported. The concentration of the libraries was determined by qPCR (Applied Biosystems Fast 7500 Real-Time PCR System) according to Illumina's protocol, substituting DyNamo HS qPCR mix (Thermo Fisher, previously New England Biolabs) for the Kappa qPCR mix. Samples were run on an updated Genome Analyzer II, or a HiSeq2000. The raw data are available from NCBI Short Reads Archive (BioProject PRJNA258398).

### Data analysis

RNA-seq reads sorted by barcodes were mapped onto MG1655 (NC_000913.2) using Bowtie 0.12.7. The mapped reads were then collated and normalized across time points by the average of the total number of spike-in reads, ssrA, ssrS, and rnpB, before normalizing by abundance at time 0 min. Within transcription units, the data were broken into 300-nucleotide (nt) bins. Transcription units that have overlapping annotations were not included in the dataset (Keseler *et al*, 2013). The half life for each fragment was obtained by fitting in Igor using the parameters.

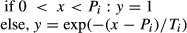
where *x* is time (min), *P*_*i*_ is the polymerase passage time (delay) for fragment *i*, and *T*_*i*_ is the lifetime of fragment *i*. Elongation rates were fitted linearly using *P*_*i*_ for each fragment by a custom Igor program. To ensure proper fitting, only transcripts longer than 1,200 nt (giving 4 data points) were analyzed. Transcription units with large uncertainty in fitting (error_rate_/rate > 1) or nonsensical results (0 < error_rate_/rate) were discarded. Other manipulations were performed in Excel. The relationship between rRNA chain elongation rate and culture temperature is known (Ryals *et al*, 1982) and may be used to estimate mRNA chain elongation rate at other temperatures.

## Supporting Information

Supplementary Information

Review Process File
