# Supplementary material for: Genome-wide study of mRNA degradation and transcript elongation in Escherichia coli
Source: Mol Syst Biol. 2015 Jan 12;11(1):781. doi: 10.15252/msb.20145794 (PMC4332155; doi:10.15252/msb.20145794)
Supplement: Supplementary file 1 [file msb0011-0781-sd1.docx]

**
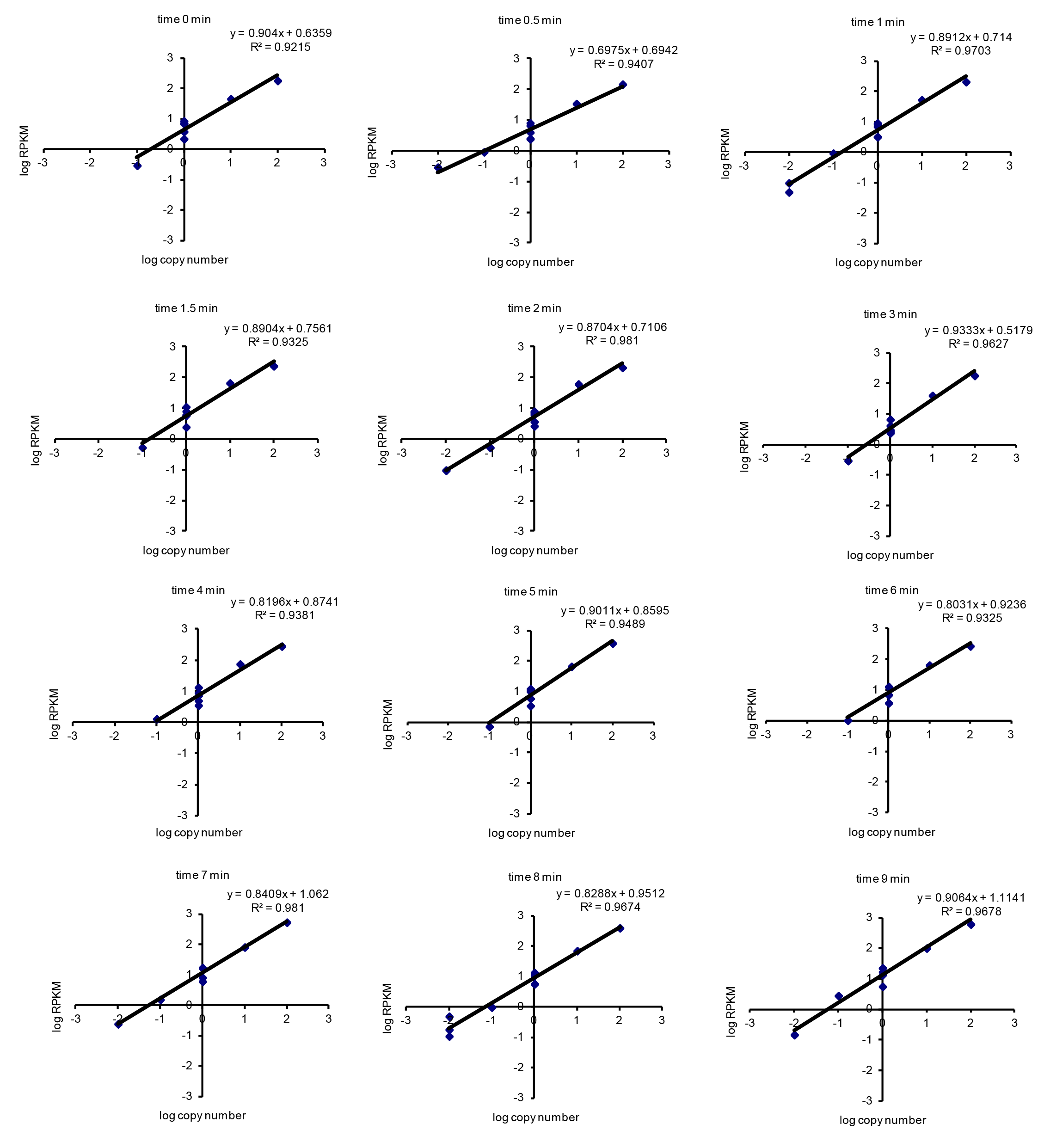
**

Supplementary Figure S1. Spike-In RNAs confirm linear correspondence between number of sequencing reads and amount of RNA in samples. The RPKM of each 300nt bin within the spike-in RNAs correspond to the number of RNAs added to the sample at each time point.
