# Supplementary material for: Genome-wide study of mRNA degradation and transcript elongation in Escherichia coli
Source: Mol Syst Biol. 2015 Jan 12;11(1):781. doi: 10.15252/msb.20145794 (PMC4332155; doi:10.15252/msb.20145794)
Supplement: Supplementary file 3 [file msb0011-0781-sd3.docx]

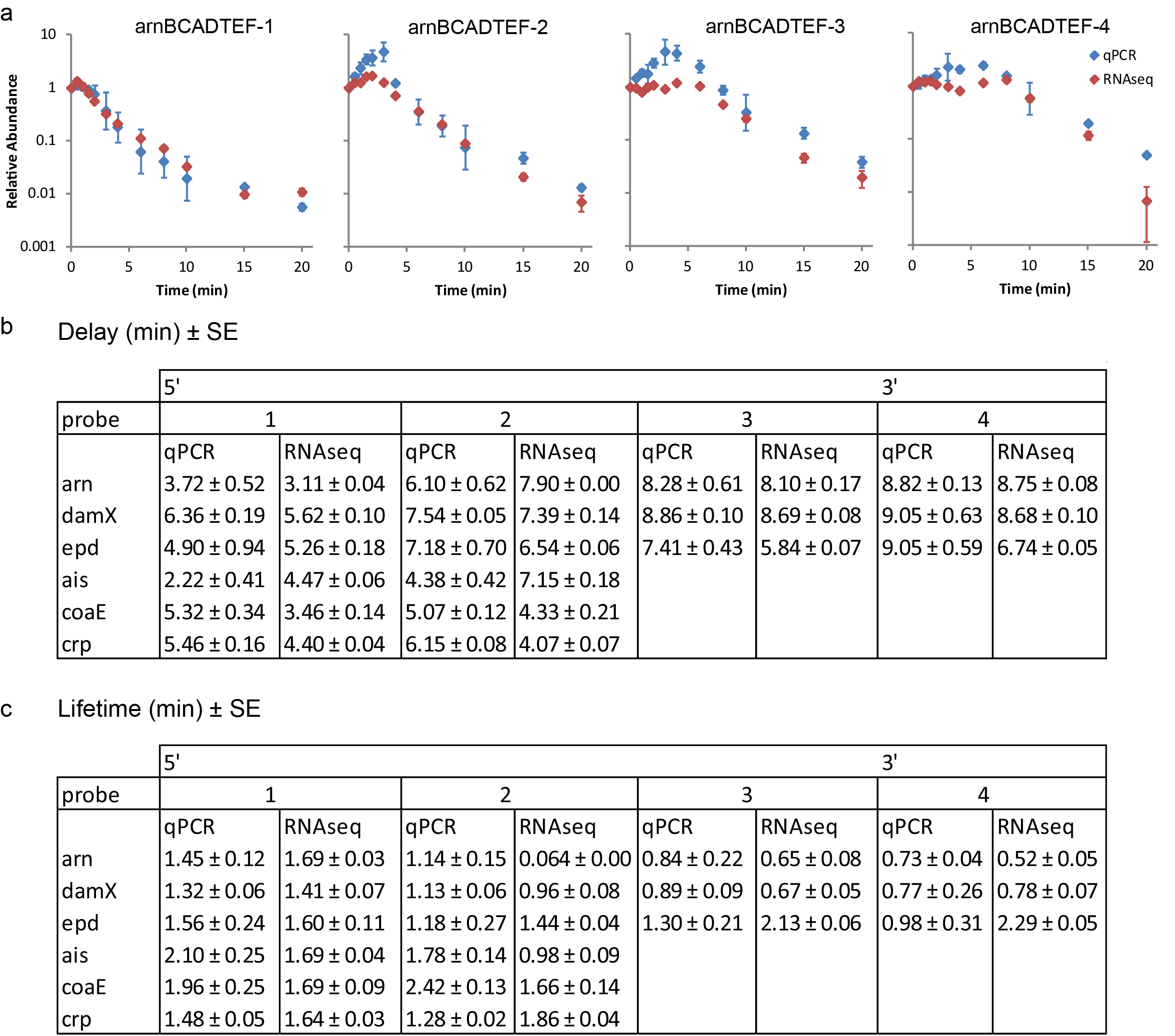


Supplementary Figure S3: Stationary phase *E.coli* RNA-seq data is confirmed by qPCR measurements. (a) Degradation traces of regions in a representative operon, arnBCADTEF, measured by qPCR and RNA-seq are similar. (b and c) Summary of fitting data from qPCR and RNA-seq degradation traces of selected transcription units. (b) The delay time fitted from qPCR and RNA-seq data are not significantly different (p = 0.38, paired Student t test). (c) The lifetimes fitted from qPCR and RNA-seq data are not significantly different (p = 0.81, paired Student t test).
