# Supplementary material for: Genome-wide study of mRNA degradation and transcript elongation in Escherichia coli
Source: Mol Syst Biol. 2015 Jan 12;11(1):781. doi: 10.15252/msb.20145794 (PMC4332155; doi:10.15252/msb.20145794)
Supplement: Supplementary file 4 [file msb0011-0781-sd4.docx]

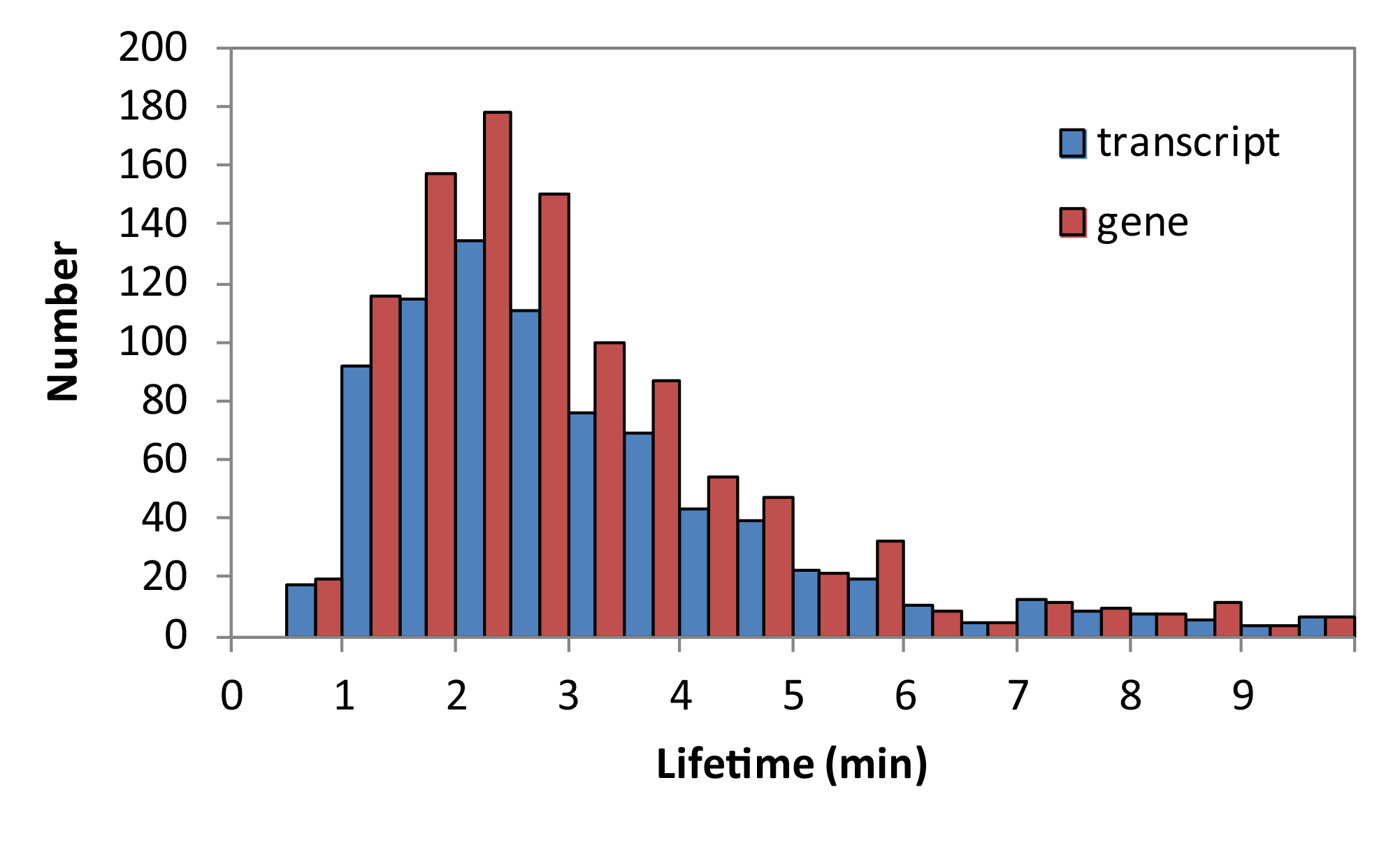


Supplementary Figure S4: Comparison of lifetime distribution of transcripts (n=847) and genes (n=1092). The lifetime distributions are not significantly different by a Kolmogonov-Smirnov test (D=0.0278, p-value=0.85).
