# Supplementary material for: Genome-wide study of mRNA degradation and transcript elongation in Escherichia coli
Source: Mol Syst Biol. 2015 Jan 12;11(1):781. doi: 10.15252/msb.20145794 (PMC4332155; doi:10.15252/msb.20145794)
Supplement: Supplementary file 5 [file msb0011-0781-sd5.docx]

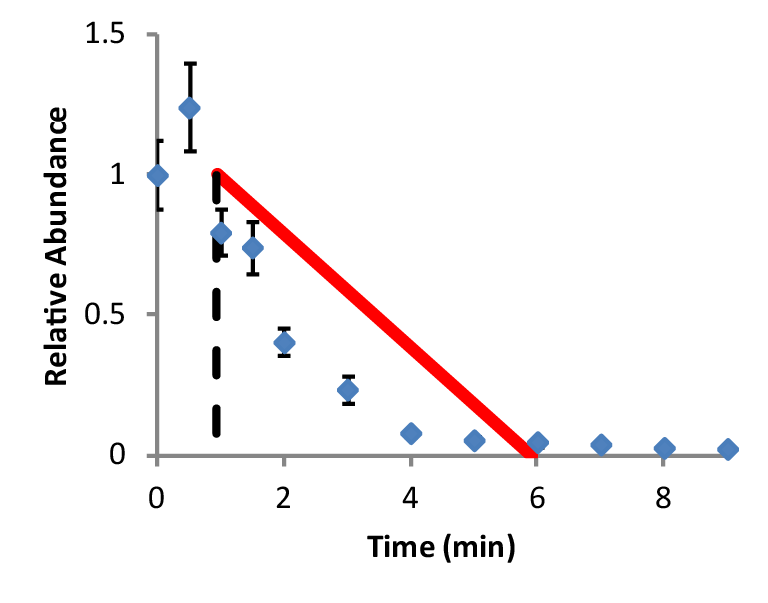


Supplementary Figure S5: Abundance of smtA-mukFEB 5’ most 300nt decays faster than predicted by post-transcriptional instantaneous degradation model (red line). We correct for the delay (dotted line) obtained from fitting.
