# Supplementary material for: Genome-wide study of mRNA degradation and transcript elongation in Escherichia coli
Source: Mol Syst Biol. 2015 Jan 12;11(1):781. doi: 10.15252/msb.20145794 (PMC4332155; doi:10.15252/msb.20145794)
Supplement: Supplementary file 15 [file msb0011-0781-sd15.docx]

**2. Supplementary**

**2.1 Linearity of sequencing data by spike-in**

Spike in RNA was prepared from phiX174 (NC001422). The genes D, F, G and H, and fragments 190 (pos 4501 - 4680) and 290 (pos 4031 - 4320) were cloned from ssDNA (New England Biolabs) with the addition of a T7 promoter. RNA was produced by *in vitro* transcription using Ambion’sMegaScript kit, according to manufacturer’s protocol. RNA integrity was verified by running on a TBE gel (BioRad). The in vitro transcribed RNAs were added to samples at the following ratios:

Gene D – 0.1 RNA/cell

Gene F – 1 RNA/cell

Gene G – 1 RNA/cell

Gene H - 0.01 RNA/cell

Fragment 190 – 100 RNA/cell

Fragment 290 – 10 RNA/cell

The number of cells in the sample was determined by counting the number of colony forming units on an LB agar plate. The RNA-seq reads were linear for the range of spike-ins (Supplementary Fig. 1).

**2.2 Reproducibility of data by qPCR (exponential phase and stationary phase)**

RNA from E.coli growing in exponential or stationary phase were collected as described in Methods, and processed. RNA abundance of two biological duplicates was measured by qPCR and compared with the corresponding RNA-seq data. The control RNAs were selected from RNA-seq data as a representative of their class – for instance RNA length, RNAP transcription elongation rate, and RNA lifetime. In some instances, traces can look similar but give different fitting results (Supplementary Fig. S2A: arnBCADTEF-1). The delay and lifetimes measured by both methods were compared by a paired Student t test, and were not significantly different, confirming the RNAseq measurement method. (Supplementary Fig. 2, 3).

**2.3 Differentially regulated genes**

The lifetimes of segments within a transcript were analyzed to identify differentially regulated transcripts. Adjacent segments on transcription units that had a lifetime different by two-fold were identified. The final list (Supplementary Table 1) was compiled after manual verification to remove noisy data or poor fits. Of the 13 identified transcripts, MalEFG have been previously reported to have differential regulation within the transcription unit(21).

**2.4 Analysis of correlation between gene function and lifetime**

Lifetimes of all 300nt bins within a transcription unit were averaged, with the exception of the 13 transcripts identified in section 2.3: Differentially regulated transcripts that were excluded. The transcript lifetime was then assigned to each gene on the transcript. Genes were grouped by gene categories obtained from J. Craig Venter Institute’s Comprehensive Microbial Resource (38). The distributions were compared using a Kolmogonov-Smirnov test.

Of the 17 gene categories, Mobile and Extrachromosomal Elements was ignored as it contained only 1 gene. 5 categories had a different distribution than the starting distribution (P<0.05), of which 4 biased towards longer lifetimes, while one was biased towards shorter lifetimes (Supplementary Fig. 5).

Comparing the distribution of lifetime of genes instead of that of transcripts, the gene lifetime distributionhas more entries with longer lifetimes (Supplementary Fig.4). The distribution of gene lifetime, compared to transcript lifetime, is not significantly different by a Kolmogonov-Smirnov test (D=0.0278, p-value = 0.85).

**Models:**

**Co-transcriptional degradation**

We will first briefly summarize the current literature related to mRNA processes, and use the concepts to build a model that will predict the mRNA degradation curves that we have observed.

mRNA synthesis is initiated by the binding of RNAP onto DNA, and chain synthesis is widely assumed to proceed at a constant speed (20). After the ribosome binding site has been transcribed, ribosomes can begin translating the mRNA even if the mRNA is not complete. Multiple ribosomes can translate a single mRNA simultaneously, and indeed polysomeswere observed for highly expressed proteins(39). It has been observed that ribosomes and RNAP activity proceed at similar speeds (18). The presence of ribosomes is proposed to be protective of mRNA – chloramphenicol, which blocks peptide chain elongation, while leaving the ribosome on the mRNA stabilizes mRNA, while kasugamycin, which prevents initiation, destabilizes mRNA (28). Decreasing the affinity of the RBS can also reduce the lifetime of an mRNA (5).

The completion of the 5’ end of the mRNA also allows degradation to commence parallel to translation. The enzyme, rppH, cleaves the 5’ triphosphate cap to leave a single phosphate, which RNaseE can now bind to the 5' monophophateand endonucleolytically degrade the mRNA (40). Both RNaseE and rppH can be the regulator of the rate determining step in mRNA degradation (41). Removal of the ribosome binding site by RNaseE prevents any further ribosome initiation, but does not interfere with ribosomes that have initiated.

To date, only 3’-to-5’ exonucleases have been found in *E.coli*. Because of the directionality of their action, these enzymes can only act after RNaseE cleavage, or after the full length RNA has been synthesized and released. The action of these downstream degradation enzymes are thought to be very rapid as degradation intermediates are not observable unless ribonuclease II and polynucleotide phosphorylase (PNPase) are knocked out (42). In *E.coli*, PNPase is thought to form a complex with RNaseE, termed the degradosome(32).

We incorporate these details into a mathematical model and introduce several parameters. Our model is concerned with the outcome of the assumption, and not the mechanism.

mRNA is synthesized at the rate of k, which is also the rate of RNAP initiation. Consistent with many modeling papers, we define k to be a first-order rate process (43; 44)

For modeling purposes, we assume that only one RNase is rate-determining, and do not distinguish between the two 5’ dependent RNases. The RNase degrades from the 5’ end, and thus does not cut between ribosomes, or between a ribosome and the RNAP.

The binding of RNase occurs on the mRNA’s 5’ end, competing with ribosome binding, and is a first-order rate process, i.e. the waiting time for RNase to bind and initiate degradation is exponentially distributed with a mean value of 1/d. d decreases with increasing [ribosome]. Similar to ribosome binding, RNase binding can occur as soon as the 5’ end is generated. Because of the sequence of events, ribosomes need to follow behind RNAP, and translocate at a similar rate to mRNA transcription.

The mRNA abundance at any position *n* consists of all the transcripts that started transcription (*n*/*v*) min prior, and may have degraded during the (*n*/*v*) min time period. Here, *v* is the velocity of transcription in nts/min. Hence the steady-state mRNA abundance at position n is

When the drug inhibiting transcription initiation is added, after time *t*

The relative abundance is

which is a delayed single exponential.

The mean waiting time for the degradation initiation of an mRNA is 1/*d*, but the mean waiting time for the complete degradation of an mRNA is 1/*d*+*L*/*v*, where *L* is the full length of the mRNA. The mean waiting time for the complete degradation of the mRNA is larger than the transcription elongation time, which explains how it is possible to have a lifetime that is shorter than the transcription elongation time – co-transcriptional degradation. Note that *d* is therate of RNA degradation typically used in the central dogma kinetic model, because after degradation initiation, protein synthesis cannot be started.

From this model, the probability of having a transcript that is degraded before the full transcript has been synthesized is 1-e*^dL^*^/^*^v^*.

**Post-transcriptional degradation: where the mRNA degrades only after it has been fully synthesized.**

This model is similar to the co-transcriptional model except for the timing of degradation initiation.

The mRNA abundance at any position *n* consists of all the transcripts that started synthesis *n*/*v* min ago from now before, and will not degrade until at least (*L*/*v*) min. Hence the steady-state mRNA abundance at position n is

When the drug inhibiting transcription initiation is added, after time t

Hence the relative abundance over time in this case would be a plateau (equilibrium) followed by a linear decay, and further followed by a single exponential decay.

The length for the linear region is L/v, which is the total time for ~seconds to minutes. It is hard to distinguish from the delayed single exponential decay in most transcripts. For instance, if *L*=1kb, *v*=25bp/s, *d*=0.5/min, then the linear decay is from 100% to 75%. For some extremely long transcript (e.x. *L*=10kb), the decay in the linear region would be much more distinct (100% to 23%). We do not see this decay pattern in longer transcripts.

**References**

35. TaniguchiY, Choi PJ, Li G-W, Chen H, Babu M, Hearn J, Emili A, Xie XS (2010) Quantifying *E.coli* proteome and transcriptome in Single Cells with Single-Molecule Sensitivity. *Science* **329***,* 533-538doi: 10.1126/science.1188308

36. Keseler IM, *et al.*(2012) EcoCyc: fusing model organism databases with systems biology.*Nucl. Ac. Res.***41** D605-D615 doi: 10.1093/nar/gks1027

37. Ryals J, Little R& BremerH (1982)Temperature Dependence of RNA Synthesis Parameters in *Escherichia coli. J. Bacteriol.***151**, 879-887

38. Davidsen T, Beck E, Ganapathy A, Montgomery R, Zafar N, Yang Q, Madupu R, Goetz P, Galinsky K, White O & Sutton G (2010) The Comprehensive Microbial Resource. *Nuc. Acids Res.***38**, D340-345 doi: 10.1093/nar/gkp912

39. Warner JR, Knopf PM & Rich A (1963) A Multiple Ribosomal Structure in Protein Synthesis. *Proc. Natl. Acad. Sci.***49**: 122–129

40. Deana A, Celesnik H & Belasco JG (2008) The bacterial enzyme RppH triggers messenger RNA degradation by 5' pyrophosphate removal.*Nature***451**(7176):355-8doi:10.1038/nature06475

41. Luciano DJ, Hui MP, Deana A, Foley PL, Belasco KJ, Belasco JG (2012) Differential Control of the Rate of 5'-End-Dependent mRNA Degradation in *Escherichia coli. J. Bacteriol.***194**, 6233-6239 doi:10.1128/JB.01223-12

42. Donovan WP& Kushner SR (1986) Polynucleotide phosphorylase and ribonuclease II are required for Cell Viability and mRNA Turnover in *Escherichia coli*”. *Proc. Nat. Ac. Sci.* **83**, 120-124

43. Bar-Even A, Paulsson J, Maheshri N, Carmi M, O’Shea E, Pilpel Y &Barkai N(2006) Noise in Protein Expression Scales With Natural Protein Abundance. *Nat. Genet.***38**, 636-43

44. Friedman N, Cai L, Xie XS (2006) “Linking Stochastic Dynamics to Population Distribution: An Analytical Framework of Gene Expression”. *Phys. Rev. Lett.* **97**, 168302

**Supplementary Methods**

**qPCR protocols and primer sequences**

1-2ug of total RNA was reverse transcribed (MMuLV, New England Biolabs) with random primers (Ambion) according to manufacturer’s protocol. Reactions were diluted 2-4 times with nuclease-free water for measurement. The following qPCR primers were used:

ssrA1.1: AAAGAGATCGCGTGGAAGCC

ssrA1.2: ACCCGCGTCCGAAATTCCTA

290_1.1: GAG ATT CTG TCT TTT CGT ATG CAG GG

290_1.2: GGG AGC ACA TTG TAG CAT TGT GCC

crp1.1: TGGCAAACCGCAAACAGACC

crp1.2: CGATGTAGTACAGCGTTTCCGC

crp2.1: CAACCAGACGCTATGACTCACC

crp2.2: GAATGCGTCCCACGGTTTCA

ais1.1: TACTGGCGCTCGCTGCAATT

ais1.2: GTGCTGCTGTGCCAGTCTGG

ais2.1: TATTGCTAAAGATAAGCGTGACG

ais2.2: CCCATCCAGATAAACTTTGCC

lpxC1.1: AGAAAGTCACCCTGACGTTACG

lpxC1.2: ACGCACAGATTTGGCATCGG

lpxC2.1: CAAACTGCTGCAGGCTGTCC

lpxC2.2: AGCTGAAGGCGCTTTGAAGG

coaE1.1: TTAACGGGAGGCATTGGCAGT

coaE1.2: CCTGACGCGCAATAATATCGGC

coaE2.1: AACTTAAGCGCACCATGCAGC

coaE2.2: TCAATGACGTCATCTGCCACG

arnBCADTEF1.1: GGAGGAACTCGCTGCAGTTAAAG

arnBCADTEF 1.2: GCGATGGCATGCTGATTTCC

arnBCADTEF2.1: CTGACCTTCGGACCACAATGG

arnBCADTEF2.2: CCATGCCGATAAACTGAGCG

arnBCADTEF3.1: TTACCTTATCGGCCTCTTCG

arnBCADTEF3.2: CGACTGATTTCCGCATAACG

arnBCADTEF4.1: GTTAGCTTTTGGCCTGGATGC

arnBCADTEF4.2: AGGCATAGGCTTTGCTTAGC

rsxABCDGE-nth1.1: AACGTTTGTGATGACGCTGG

rsxABCDGE-nth1.2: CAGAATAAATGCCAGGGTGC

rsxABCDGE-nth2.1: CAC ATG GCG GGC GTT CAT C

rsxABCDGE-nth2.2: AGCGATTGCTTCGCCAGTCA

rsxABCDGE-nth3.1: CTACAGCGGTATTCTGGCGG

rsxABCDGE-nth3.2: GCGAATCGCTTTCTGCCATA

rsxABCDGE-nth4.1: ATCGTACTCAATTTGCGCCGG

rsxABCDGE-nth4.2: CCCGTGCAGGATCAACCAAT

epd-pgk-fbaA1.1: GTGCTTTGTATGAATCCGGACG

epd-pgk-fbaA1.2: TTCAACAAATGCGCCATGCC

epd-pgk-fbaA2.1: GATGACCGATCTGGATCTTGCTG

epd-pgk-fbaA2.2: CGCGTCGCTGGTTACTTTCC

epd-pgk-fbaA3.1: GGTTGGCGTGTTCGAATTCC

epd-pgk-fbaA3.2: ATTGCTGCCAGAGTGTCGCC

epd-pgk-fbaA4.1: CATCGATACCGATACCCAATGG

epd-pgk-fbaA4.2: TTCGCCTTTCGGGTTACCCA

damX-dam-rpe-gph-trpS1.1: AACCAGAAGACGAGCTGAAACC

damX-dam-rpe-gph-trpS1.2: AATTGATCTGCGGTTCGCCA

damX-dam-rpe-gph-trpS2.1: TAAACGGCATTTGCCCAAGG

damX-dam-rpe-gph-trpS2.2: GATCAGGTCGCTATTGATATCGG

damX-dam-rpe-gph-trpS3.1: CTTGCTGCTGCGGTAGATATGG

damX-dam-rpe-gph-trpS3.2: TCTGCGCCGTTACCAATCCA

damX-dam-rpe-gph-trpS4.1: GAAAGGTGAAGTGGCTGATGC

damX-dam-rpe-gph-trpS4.2: CAGGAAGGCTTCATCGTTGC

rfe1.1: aaccaaacttccgcaaacgtcacc

rfe1.2: AAAACAAGCA CACCGGCACA AGC

rffH1.1: actcacgacagcctgattgaagc

rffH1.2: TTCGCTAATG AACTGGCAGC ACG

rffC1.1: aggtgaagttgatttggcgctacc

rffC1.2: CGAAAACGGC TTTGCGCAAA TGC

rffM1.1: ccaaagcaggagatcatcatgcg

rffM1.2: AGCGTTTGCC AGATTTTCGG TGC

**Supplementary Tables Legends**

**Supplementary Table S1**: Delay and lifetime fitting results for 847 transcription units measured at exponential phase. RNA abundance measured by RNA-seq was broken into 300-nt bins and fitted to extract the delay (column header Posi) and lifetime and are reported with fitting error.

**Supplementary Table S2**: Delay and lifetime fitting results for 1259 transcription units measured at stationary phase. RNA abundance measured by RNA-seq was broken into 300-nt bins and fitted to extract the delay (column header Posi) and lifetime and are reported with fitting error.

**Supplementary Table S3**: Transcripts that do not have a constant lifetime. A subset of transcripts from Supplementary Table S1 that do not have a constant lifetime (defined as a 2-fold difference in lifetime of adjacent fragments) are reported.

**Supplementary Table S4**: Average lifetime of 847 transcripts measured at exponential phase. The lifetime of the 300-nt bins are averaged to report a single average lifetime with standard deviation for each transcript.

**Supplementary Figure S5**: Distribution of lifetime by gene function. The distributions of lifetimes in each gene functional category were compared to the general population using the Kolmogorov-Smirnov test. 5 gene functional groups had a different distribution with a P-value < 0.05 (highlighted in green).

**Supplementary Table S6**: Elongation rate fitting results for 263 transcripts measured at exponential phase. 263 measured transcripts were longer than 1200-nt, and could be fitted for elongation rate. The fitting error is reported.

**Supplementary Table S7**: Average lifetime of 1259 transcripts measured at stationary phase. The lifetime of the 300-nt bins are averaged to report a single average lifetime with standard deviation for each transcript.

**Supplementary Table S8**: Elongation rate fitting results for 423 transcripts measured at stationary phase. 263 measured transcripts were longer than 1200-nt, and could be fitted for elongation rate. The fitting error is reported.

**Supplementary Table S9**: Synthesis time to 5’ end lifetime ratios for 263 transcripts measured at exponential phase. For the 263 transcripts with a measured elongation rate at exponential phase, the complete transcript synthesis time was calculated and taken as a ratio of the 5’ end lifetime. This allows us to estimate the prevalence of co-transcriptional degradation.
