# Supplementary material for: Genome-wide study of mRNA degradation and transcript elongation in Escherichia coli
Source: Mol Syst Biol. 2015 Jan 12;11(1):781. doi: 10.15252/msb.20145794 (PMC4332155; doi:10.15252/msb.20145794)
Supplement: Supplementary file 16 [file msb0011-0781-sd16.pdf]

Genome-wide study of mRNA degradation and transcript elongation in Escherichia coli

Huiyi Chen, Katsuyuki Shiroguchi, Hao Ge, Xiaoliang Sunney Xie

Corresponding author: Xiaoliang Sunney Xie, Harvard University

Review timeline:

Submission date:	25 September 2014
Editorial Decision:	04 November 2014
Revision received:	03 December 2014
Editorial Decision:	05 December 2014
Revision received:	07 December 2014
Accepted:	13 December 2014

Editor: Maria Polychronidou

Transaction Report:

(Note: With the exception of the correction of typographical or spelling errors that could be a source of ambiguity, letters and reports are not edited. The original formatting of letters and referee reports may not be reflected in this compilation.)

1st Editorial Decision 04 November 2014

Thank you again for submitting your work to Molecular Systems Biology. We have now heard back from the three referees who agreed to evaluate your manuscript. As you will see below, the reviewers acknowledge that the presented findings are interesting. However, they raise a series of concerns and make suggestions for modifications, which we would ask you to address in a revision of this work. The referees' recommendations are clear and therefore there is no need to repeat their comments.

Reviewer #1:

mRNA degradation is an important means by which gene expression is controlled in all organisms. Previous studies in E. coli have measured the half-lives of mRNAs one at a time or transcriptome-wide, the latter experiments having involved the use of microarrays. In the present study, the lifetimes of segments of E. coli mRNAs were measured transcriptome-wide by RNA-seq after inhibiting RNA polymerase with rifampicin or streptolydigin. The authors report a lag in the disappearance of the promoter-distal regions of longer transcripts following rifampicin addition, which represents the time required for nascent transcripts to extend past those regions. As a result, they are able to calculate both transcription elongation rates and mRNA degradation rates for many genes and operons in E. coli. The range of elongation rates that they report are consistent with previous studies. The average mRNA half-life that they measure (2.4 min) is shorter than that previously reported on the basis of microarray studies (6 min) because the latter studies did not correct for the lag in the observable onset of decay after rifampicin addition. The authors also observe that, with some exceptions, the rate constant for decay typically is similar at all positions within a given transcript, implying that the rate determining step in decay usually governs the rate of

© European Molecular Biology Organization

degradation of the entire transcript. Finally, they identify several long transcripts for which the decay of the 5'-proximal portion precedes the synthesis of the 3'-proximal portion (i.e., for which decay begins before transcription is complete). Although a number of these conclusions (elongation rates, half-lives, uniform rate of decay throughout a transcript, co-transcriptional degradation) have previously been reached for individual transcripts or by other methods, the scale of this study makes it the most comprehensive to date.

Additional points:

1) Page 3, paragraph 4. The authors should explain how the information in Figure 1a enables them to conclude that "there is less RNA on the 5' ends compared to 3' ends of RNA transcription units, relative to the zero minute time point."

2) Page 5, paragraph 4. The statement that "it can also happern in the case of trans-translation", where "it" appears to refer to "degradation of the 5' end of an RNA before the synthesis of its 3' end", is mistaken, as trans-translation is a cellular mechanism for resolving problems with translation termination that can arise when the 3' portion of an mRNA translational unit decays before the ribosome-bound 5' portion. As such, it is evidence for co-translational degradation, not co-transcriptional degradation.

3) Page 6, paragraphs 4-7. The discussion of the co-transcriptional and post-transcriptional degradation models requires clarification, as it is not clear how some of the conclusions (linear decay of RNA, dependence on the binding rate rather than the cleavage rate of a ribonuclease, relative abundance of the 5' end of RNA, etc.) were reached.

4) Page 8, paragraph 2. "The prevalence of a single exponential decay along transcripts suggests 5' to 3' end directed RNA degradation ... ." Does this conclusion apply only to transcripts for which degradation of the 5' end precedes synthesis of the 3' end or to all transcripts, irrespective of whether the entire transcript is usually synthesized before decay begins? It's not clear how this conclusion could apply to transcripts in the latter category.

5) Figures. It is almost impossible to make sense of many of the figures without carefully reading the figure legends. It would help if more information were provided in the figures themselves in order to make them a bit more self-explanatory.

Reviewer #2:

The manuscript by Chen et al probes position-dependent mRNA degradation in E. coli by performing RNA-seq upon inhibiting transcription initiation. The authors identified an initial delay in degradation, which is attributed to the time it takes for the last polymerase to produce the corresponding segment of the transcript. After the initial delay, the degradation follows single exponential decay. The decay constant varies across transcripts, but is similar among different regions of the same transcript. This work provides the first genome-wide measurement of transcription elongation rates as well as high-resolution mRNA degradation rates. The observation that the majority of mRNAs decay as a first-order process is important and novel.
1. The experiments are technically sound, and the analysis of sequencing results is done properly. It is very important that the data is publically available and easily accessible, and currently this is not true. This issue can be addressed by including supplementary files with RNA half lives. It is also important that the manuscript contain sufficient detail that the procedure can be repeated.
2. In relation to the mechanism of mRNA degradation, I encourage the authors to discuss the implications of this work for our understanding of the decay pathway. This discussion will have a meaningful impact on the field of post-transcriptional regulation in bacteria.
3. The speed of RNAP is synchronized with the speed of ribosome, both of which are highly dependent on temperature. In comparison with other literature values of transcription elongation rate, the authors stated "Many other elongation rates have been measured by cloning the gene of interest onto a plasmid behind an inducible promoter (20; 18). However, it has shown that plasmid-based measurements of elongation rates can be changed by varying the concentration of IPTG."However, many of these measurements are also done at different temperatures. So the difference may simply due to temperature rather than as a result of IPTG concentration.

Reviewer #3:

In this study, the authors have investigated in depth the response of transcripts following the addition of the antibiotic rifampicin to the media, that is widely used to stop the transcription and observe the stability of the RNA of interest. The authors show experimentally that the time lag before the onset of transcript decay arises from RNA polymerases that are actively transcribing the RNA, and cannot be targeted by the antibiotic. This simple observation has enabled the authors to model simultaneously the RNA polymerase elongation rates and RNA degradation rates. The experimental work is at a high standard and is well controlled. The authors have used RNA sequencing at frequent time intervals following treatment with rifampicin to follow the decay of transcripts. The lifetime of transcripts in E. coli has been estimated in earlier studies based on inhibition of RNA polymerase by the antibiotic rifampicin, but these have not included the critical correction for the lag. Using this critical correction, and from the depth and coverage of their data for the entire E. coli genome, the authors have made many insightful findings. One surprising finding is that RNA degradation rates do not vary by more than 10-fold for most transcripts, while RNA abundance does, and this scales linearly with translation rates, regardless of growth conditions - exponential versus stationary phase. Another important finding is that decay rates do not seem to vary along many transcripts generally. The authors propose that post-transcriptional regulation is not as important as rates of synthesis in defining RNA abundance. This is a very nice experimental study, and there are only a few minor comments and suggestions that hopefully will be helpful for the authors to consider.

1. Do these RNAs also have equal access to the translation machinery, i.e, do the abundances correlate with ribosome occupancy from available ribosome profiling data ?

2. Abstract, first line. It might be viewed as too simplistic a view "same cellular compartment" The statement is true but glosses over the interesting implications that some of the degradative machinery is in an honorary sub-compartment, because the enzymes are membrane associated (such as RNase E or RNase Y in many species).

3. It might be helpful for the non-expert reader to understand the principles of the experiment if the authors explain it in more transparent way, for example, what Spike-in RNAs are, what are the 2 phases they have in mind in the last paragraph of discussion.

4. Would it be possible to state how many RNAs with particular feature were found rather than giving one or two examples? (e.g. smtA-mukFEB operon case)

5. The authors do not comment about decay rates for non-coding RNA, such as sRNAs or 6S RNA.

6. Page 1 intro line 2 "spatial arrangement of RNA" might confuse reader - more precise might be "spatial localisation"

7. Page 1 intro line 12 "increased the number .." this and following sentences seem to overfocus on the the history of high throughout approaches. Perhaps it is not necessary to go into so much detail, which is distracting to the general points.

8. Last sentences of section in result section 'Simultaneous measurement of RNA chain elongation and degradation genome wide using rifampicin' not immediately clear how the 30 seconds is deduced.

9. 'Global behavior of RNA degradation section, line 5 typo poly-cistronic

10. The authors might wish to reference a review by George Mackie in Nature reviews in microbiology, which describes the consequences of membrane compartmentalisation of ribonucleases. This compartmentalisation will have bearing on the models for co-transcriptional decay.

11. The experiments were done at 30 C. Does the rifampicin lag at different temperature?

1st Revision - authors' response 03 December 2014

Reviewer #1:

mRNA degradation is an important means by which gene expression is controlled in all organisms. Previous studies in E. coli have measured the half-lives of mRNAs one at a time or transcriptome-wide, the latter experiments having involved the use of microarrays. In the present study, the lifetimes of segments of E. coli mRNAs were measured transcriptome-wide by RNA-seq after inhibiting RNA polymerase with rifampicin or streptolydigin. The authors report a lag in the disappearance of the promoter-distal regions of longer transcripts following rifampicin addition, which represents the time required for nascent transcripts to extend past those regions. As a result, they are able to calculate both transcription elongation rates and mRNA degradation rates for many genes and operons in E. coli. The range of elongation rates that they report are consistent with previous studies. The average mRNA half-life that they measure (2.4 min) is shorter than that previously reported on the basis of
microarray studies (6 min) because the latter studies did not correct for the lag in the observable onset of decay after rifampicin addition. The authors also observe that, with some exceptions, the rate constant for decay typically is similar at all positions within a given transcript, implying that the rate determining step in decay usually governs the rate of degradation of the entire transcript. Finally, they identify several long transcripts for which the decay of the 5'-proximal portion precedes the synthesis of the 3'-proximal portion (i.e., for which decay begins before transcription is complete). Although a number of these conclusions (elongation rates, half-lives, uniform rate of decay throughout a transcript, co-transcriptional degradation) have previously been reached for individual transcripts or by other methods, the scale of this study makes it the most comprehensive to date.

Additional points:

1) Page 3, paragraph 4. The authors should explain how the information in Figure 1a enables them to conclude that "there is less RNA on the 5' ends compared to 3' ends of RNA transcription units, relative to the zero minute time point."

We thank the reviewer's suggestion. We have included more details on the data analysis in the Results section (page 3, paragraph 4) to clarify how Fig. 1a was made, and thus how we can conclude that relative to the zero minute time point, there is less RNA on the 5' ends compared to the 3' ends of RNA transcription units.

2) Page 5, paragraph 4. The statement that "it can also happern in the case of trans-translation", where "it" appears to refer to "degradation of the 5' end of an RNA before the synthesis of its 3' end", is mistaken, as trans-translation is a cellular mechanism for resolving problems with translation termination that can arise when the 3' portion of an mRNA translational unit decays before the ribosome-bound 5' portion. As such, it is evidence for co-translational degradation, not co-transcriptional degradation.

We agree with the reviewer's comment. We removed this sentence from the manuscript.

3) Page 6, paragraphs 4-7. The discussion of the co-transcriptional and post-transcriptional degradation models requires clarification, as it is not clear how some of the conclusions (linear decay of RNA, dependence on the binding rate rather than the cleavage rate of a ribonuclease, relative abundance of the 5' end of RNA, etc.) were reached.

We thank the reviewer's suggestion. We explained the assumptions of the model in page 6, paragraph 3, and described the results of the modeling in paragraphs 4-6. The actual mathematical solution was put in the supplementary for interested readers. To make the fact that our conclusions

are based on math more apparent, we have described the mathematical solution before explaining the solution in words page 6, paragraphs 5 and 6 .

4) Page 8, paragraph 2. "The prevalence of a single exponential decay along transcripts suggests 5' to 3' end directed RNA degradation ... ." Does this conclusion apply only to transcripts for which degradation of the 5' end precedes synthesis of the 3' end or to all transcripts, irrespective of whether the entire transcript is usually synthesized before decay begins? It's not clear how this conclusion could apply to transcripts in the latter category.

We thank the reviewer for pointing out the confusion. In our paper, we assumed that there is only one mechanism for all transcripts, but it is stochastic: either the degradation of the 5' end is allowed to precede the synthesis of the 3' end, or all the transcripts should always decay after the 3'end is synthesized. Our paper concludes that the previous category of mechanism is consistent with the data, while the latter one is not.

Even in the case where the 5’ end is allowed to degrade before synthesis of the 3’ end, there are two categories of mRNAs in the cell due to stochasticity, i.e. the degradation occurs before or after the 3'end is synthesized. But it does not mean there are two categories of mechanisms. There is only one single mechanism.

Now we change the sentence to clarify as follow:“The observed constant exponential lifetime along transcripts suggests 5 ' to 3‘ directed RNA degradation, and supports the mechanism of co-transcriptional degradation for each transcription unit." (Page 8, paragraph 2)

5) Figures. It is almost impossible to make sense of many of the figures without carefully reading the figure legends. It would help if more information were provided in the figures themselves in order to make them a bit more self-explanatory.

We thank the reviewer’s suggestion. We have modified and added more labels to the figures (Fig. 1, 2, 3, and 4) so that they can be better understood without reading the legends.

Reviewer #2:

The manuscript by Chen et al probes position-dependent mRNA degradation in E. coli by performing RNA-seq upon inhibiting transcription initiation. The authors identified an initial delay in degradation, which is attributed to the time it takes for the last polymerase to produce the corresponding segment of the transcript. After the initial delay, the degradation follows single exponential decay. The decay constant varies across transcripts, but is similar among different regions of the same transcript. This work provides the first genome-wide measurement of transcription elongation rates as well as high-resolution mRNA degradation rates. The observation that the majority of mRNAs decay as a first-order process is important and novel.

1. The experiments are technically sound, and the analysis of sequencing results is done properly. It is very important that the data is publically available and easily accessible, and currently this is not true. This issue can be addressed by including supplementary files with RNA half lives. It is also important that the manuscript contain sufficient detail that the procedure can be repeated.
We thank the reviewer's suggestion. We have submitted additional Excel files with results of the fittings and analyses on the journal’s website as part of the Supplementary and Source Data.

2. In relation to the mechanism of mRNA degradation, I encourage the authors to discuss the implications of this work for our understanding of the decay pathway. This discussion will have a meaningful impact on the field of post-transcriptional regulation in bacteria.

We thank the reviewer's suggestion. We did not specify the decay pathway much since our measurements and modeling cannot tell apart players in the pathway. However, we agree with the

reviewer's point, and we list the possible relevant players for the rate-limiting step in explaining the model (page 6 paragraph 3).

3. The speed of RNAP is synchronized with the speed of ribosome, both of which are highly dependent on temperature. In comparison with other literature values of transcription elongation rate, the authors stated "Many other elongation rates have been measured by cloning the gene of interest onto a plasmid behind an inducible promoter (20; 18). However, it has shown that plasmid-based measurements of elongation rates can be changed by varying the concentration of IPTG."However, many of these measurements are also done at different temperatures. So the difference may simply due to temperature rather than as a result of IPTG concentration.

We thank the reviewer for raising an important point. Certainly, we are aware that temperature contributes to the elongation rate, and have taken care to compare measurements only when they were performed at comparable temperatures, which is why we did not compare the other references. In our statement "However, it has been shown that plasmid-based measurements of elongation rates can be changed by varying the concentration of IPTG (24)." which the reviewer referred in the comment above, we specifically referred to reference 24 to support the idea that IPTG concentration can alter the elongation rate. In this paper (reference 24), the experiment was done at one temperature, and the authors altered the number of RNAPs by using different concentrations of IPTG in a lac promoter construct, and showed a difference in elongation rate.

Reviewer #3:

In this study, the authors have investigated in depth the response of transcripts following the addition of the antibiotic rifampicin to the media, that is widely used to stop the transcription and observe the stability of the RNA of interest. The authors show experimentally that the time lag before the onset of transcript decay arises from RNA polymerases that are actively transcribing the RNA, and cannot be targeted by the antibiotic. This simple observation has enabled the authors to model simultaneously the RNA polymerase elongation rates and RNA degradation rates. The experimental work is at a high standard and is well controlled. The authors have used RNA sequencing at frequent time intervals following treatment with rifampicin to follow the decay of transcripts. The lifetime of transcripts in E. coli has been estimated in earlier studies based on inhibition of RNA polymerase by the antibiotic rifampicin, but these have not included the critical correction for the lag. Usingthis critical correction, and from the depth and coverage of their data for the entire E. coli genome, the authors have made many insightful findings. One surprising finding is that RNA degradation rates do not vary by more than 10-fold for most transcripts, while RNA abundance does, and this scales linearly with translation rates, regardless of growth conditions - exponential versus stationary phase. Another important finding is that decay rates do not seem to vary along many transcripts generally. The authors propose that post-transcriptional regulation is not as important as rates of synthesis in defining RNA abundance. This is a very nice experimental study, and there are only a few minor comments and suggestions that hopefully will be helpful for the authors to consider.

1. Do these RNAs also have equal access to the translation machinery, i.e, do the abundances correlate with ribosome occupancy from available ribosome profiling data ?

We thank the reviewer for this thoughtful question. One way to check whether RNAs have equal access to translation machinery is to look at the correlation of ribosome occupancy and RNA abundance as the reviewer suggested. This data is available from Li et al.'s paper (Cell 2014 157(3): 624-635), Supplementary Table 4. There is no correlation between translation efficiency, the term used by the paper, and abundance.

Abundance is determined by both synthesis and degradation rates. We expect ribosome occupancy to affect the degradation rates. However, we find in our preliminary analysis that degradation rates are not correlated with ribosome occupancy. While it seems surprising to find that ribosome protection does not determine RNA lifetime, this could be explained by the fact that lifetimes are

determined by many factors, including RNases having different affinities for the different RNAs.

2. Abstract, first line. It might be viewed as too simplistic a view "same cellular compartment" The statement is true but glosses over the interesting implications that some of the degradative machinery is in an honorary sub-compartment, because the enzymes are membrane associated (such as RNase E or RNase Y in many species).

We thank the reviewer's suggestion. After deep consideration, we have decided to keep the current abstract since we think there are many readers who may be unaware of the evolution in thinking about compartmentalization in prokaryotes. However since we think that this point is important, we mention this in the Results and Discussion.(page 6, paragraph 3 and page 8, paragraph 2).

3. It might be helpful for the non-expert reader to understand the principles of the experiment if the authors explain it in more transparent way, for example, what Spike-in RNAs are, what are the 2 phases they have in mind in the last paragraph of discussion.

We thank the reviewer's suggestion, and modified the manuscript accordingly (page 3, paragraph 4 and page 8, paragraph 2).

4. Would it be possible to state how many RNAs with particular feature were found rather than giving one or two examples? (e.g. smtA-mukFEB operon case)

We thank the reviewer for his suggestion. We have included the complete list of RNAs with particular features (Supplementary Table 3: List of RNA with 2-fold difference in lifetime along transcript; Supplementary Table 8: Transcription units with synthesis and degradation times).

5. The authors do not comment about decay rates for non-coding RNA, such as sRNAs or 6S RNA.

We thank the reviewer's for pointing this out. Our data includes non-coding RNAs. Our genome-wide experiment simultaneously measured the decay of all transcription units with non-overlapping annotation, with a minimum length of 300 nt. The decay rates of transcription units with non-coding RNAs that fit this criteria are reported in Supplementary Tables 1 and 5. We added explanation of this in the table.

6. Page 1 intro line 2 "spatial arrangement of RNA" might confuse reader - more precise might be "spatial localisation"

We thank the reviewer's suggestion, and modified the manuscript accordingly (page 1 intro line 2).

7. Page 1 intro line 12 "increased the number .." this and following sentences seem to overfocus on the the history of high throughout approaches. Perhaps it is not necessary to go into so much detail, which is distracting to the general points.

We thank the reviewer's suggestion. We have removed details from the history of high throughput approaches, and focused on the advantages of system-wide data on analysis and modeling (Introduction, paragraph 2).

8. Last sentences of section in result section 'Simultaneous measurement of RNA chain elongation and degradation genome wide using rifampicin' not immediately clear how the 30 seconds is deduced.

We agree with the reviewer's point, and removed the sentences.

© European Molecular Biology Organization

9. 'Global behavior of RNA degradation section, line 5 typo poly-cistronic

We thank the reviewer's indication, and modified the manuscript accordingly.

10. The authors might wish to reference a review by George Mackie in Nature reviews in microbiology, which describes the consequences of membrane compartmentalisation of ribonucleases. This compartmentalisation will have bearing on the models for co-transcriptional decay.

We thank the reviewer's suggestion. We agree that George Mackie's review raises relevant points about modeling RNA degradation and we have accordingly inserted discussions on sub-cellular compartmentalization in the Discussion (page 8, paragraph 2). We designed an experiment to obtain sufficient data to make a quantitative model to understand degradation. We believe that this basic model will contribute to understanding RNA degradation in a more quantitative and testable manner. As the reviewer implies, this model may be modified by quantitative data from experiments probing the contribution of sub-cellular compartmentalization on the kinetics of RNA processing.

11. The experiments were done at 30 C. Does the rifampicin lag at different temperature?

In preliminary experiments, we have observed a lag in degradation when using rifampicin at 37C as well.

2nd Editorial Decision 05 December 2014

Thank you again for submitting your work to Molecular Systems Biology. We are now satisfied with the modifications made and we think that the study is suitable for publication.

Before we formally accept the manuscript, we would like to ask you to address the following issues:
- In line with comment #4 of reviewer #3 on the previous version of the manuscript, we would ask you to include a sentence in the main text, mentioning that the decay rates of non-coding transcripts were measured as well and referring to the tables containing the relevant data.

Thank you for submitting this paper to Molecular Systems Biology.

1st Revision - authors' response 07 December 2014

Response to editorial comments
- In line with comment #4 of reviewer #3 on the previous version of the manuscript, we would ask you to include a sentence in the main text, mentioning that the decay rates of non-coding transcripts were measured as well and referring to the tables containing the relevant data.

We have included a sentence in the main text to mention that non-coding RNA were measured, and referred to the tables with the relevant data (page 3, paragraph 4).

© European Molecular Biology Organization
